# Mediterranean Marine Mammals: Possible Future Trends and Threats Due to Mercury Contamination and Interaction with Other Environmental Stressors

**DOI:** 10.3390/ani14162386

**Published:** 2024-08-17

**Authors:** Roberto Bargagli, Emilia Rota

**Affiliations:** Department of Physics, Earth and Environmental Sciences, University of Siena, Via P.A. Mattioli 4, 53100 Siena, Italy; roberto.bargagli@unisi.it

**Keywords:** Mediterranean Sea, cetaceans, monk seal, mercury, climate change, organic contaminants, interactions, possible cumulative effects

## Abstract

**Simple Summary:**

Marine mammals in the Mediterranean are among the most exposed globally to mercury, persistent organic pollutants (legacy or emerging), and micro- and nanoplastics. Moreover, the semi-enclosed basin is highly sensitive to climate change, with enhanced water warming, possible changes in primary productivity and food webs, and a predictable increasing remobilization of soil- and sediment-bound persistent pollutants. In this context, this review discusses the physico-chemical and bio-ecological processes driving the exceptional bioaccumulation of mercury in Mediterranean cetaceans and possible future trends. Marine mammals detoxify and store Hg in the liver and other organs as insoluble crystals, but this process can deplete the biological pool of an essential element such as selenium and expose adult cetaceans to infectious and autoimmune diseases. Although difficult to assess, an even more serious threat to Mediterranean cetaceans may be the concomitant and remarkable accumulation of synthetic organic pollutants. The NW Mediterranean has been declared a cetacean sanctuary, yet the whales and dolphins living there tend to have the highest concentrations of most persistent pollutants. Therefore, we emphasize the adoption of all available measures to mitigate anthropogenic pressures and studies to evaluate the biological effects of chronic and concurrent exposure to persistent environmental pollutants.

**Abstract:**

Despite decreasing anthropogenic mercury (Hg) emissions in Europe and the banning and restriction of many persistent organic pollutants (POPs) under the Stockholm Convention, Mediterranean marine mammals still have one of the highest body burdens of persistent pollutants in the world. Moreover, the Mediterranean basin is one of the most sensitive to climate change, with likely changes in the biogeochemical cycle and bioavailability of Hg, primary productivity, and the length and composition of pelagic food webs. The availability of food resources for marine mammals is also affected by widespread overfishing and the increasing number of alien species colonizing the basin. After reporting the most recent findings on the biogeochemical cycle of Hg in the Mediterranean Sea and the physico-chemical and bio-ecological factors determining its exceptional bioaccumulation in odontocetes, this review discusses possible future changes in the bioavailability of the metal. Recent ocean–atmosphere–land models predict that in mid-latitude seas, water warming (which in the Mediterranean is 20% faster than the global average) is likely to decrease the solubility of Hg and favor the escape of the metal to the atmosphere. However, the basin has been affected for thousands of years by natural and anthropogenic inputs of metals and climate change with sea level rise (3.6 ± 0.3 mm year^−1^ in the last two decades), and the frequency of extreme weather events will likely remobilize a large amount of legacy Hg from soils, riverine, and coastal sediments. Moreover, possible changes in pelagic food webs and food availability could determine dietary shifts and lower growth rates in Mediterranean cetaceans, increasing their Hg body burden. Although, in adulthood, many marine mammals have evolved the ability to detoxify monomethylmercury (MMHg) and store the metal in the liver and other organs as insoluble HgSe crystals, in Mediterranean populations more exposed to the metal, this process can deplete the biological pool of Se, increasing their susceptibility to infectious diseases and autoimmune disorders. Mediterranean mammals are also among the most exposed in the world to legacy POPs, micro- and nanoplastics, and contaminants of emerging interest. Concomitant exposure to these synthetic chemicals may pose a much more serious threat than the Se depletion. Unfortunately, as shown by the literature data summarized in this review, the most exposed populations are those living in the NW basin, the main feeding and reproductive area for most Mediterranean cetaceans, declared a sanctuary for their protection since 2002. Thus, while emphasizing the adoption of all available approaches to mitigate anthropogenic pressure with fishing and maritime traffic, it is recommended to direct future research efforts towards the assessment of possible biological effects, at the individual and population levels, of chronic and simultaneous exposure to Hg, legacy POPs, contaminants of emerging interest, and microplastics.

## 1. Introduction

The Mediterranean Sea has a long history of mercury (Hg) inputs from natural and anthropogenic sources [1,2], and it has been known since the 1970s that its resident long-lived carnivorous species, particularly tuna, swordfish, and dolphins, have much higher Hg concentrations than similarly sized Atlantic species (i.e., the so-called “Mediterranean Hg anomaly”) [3,4,5]. Although declining anthropogenic emissions of gaseous elemental Hg (Hg°) in Europe, coupled with increased Hg° evasion from warming surface seawater, may be reducing the metal’s bioavailability to Mediterranean organisms [6], the data collected over the last 40 years show that Hg levels in several predatory species still exceed European Union (EU) regulatory thresholds [7]. The mercury concentrations in Mediterranean cetaceans are the highest in the world and are often above levels that could cause potential adverse effects [8]. Cetaceans are long-lived apex predators that consume a wide variety of prey and, unlike fish [9], do not have gills for the direct absorption/excretion of essential and potentially toxic elements. They can intake Hg via the placenta and lactation, from water and air through the skin and lungs, but their prey is by far the most important source [10]. The liver is involved in the homeostatic regulation of ingested essential elements and the detoxification of non-essential elements [11] and is, therefore, the target organ for Hg. However, unlike terrestrial mammals, which are mainly exposed to inorganic Hg (iHg) and detoxify the metal with metallothioneins (MTs), marine mammals are mainly exposed to the neurotoxic monomethylmercury (MMHg) and have evolved the ability to demethylate and sequester the metal with selenium (Se) in a non-toxic compound: HgSe (mercuric selenide, also known as tiemannite) [12]. Therefore, total Hg concentrations in the organs and tissues of Mediterranean dolphins are one to two orders of magnitude higher than those of tuna or swordfish, which have quite similar trophic levels and average life spans [13,14].

In 2008, the European Union adopted the Marine Strategy Framework Directive (2008/56/EC) to maintain or restore marine ecosystems, and since 1992, marine mammals, as “charismatic megafauna” at the top of the marine food webs, have been strictly protected (EU Habitats Directive, Annex IV) and play a priority role in the monitoring and management of the marine environment. The Mediterranean populations are genetically distinct from their Atlantic counterparts and are seriously threatened by anthropogenic activities at sea (fishing and entanglement in fishing gear, collisions, or noise pollution from ships) and by environmental pollutants such as Hg, persistent organic pollutants (POPs), and marine litter [8,15,16,17].

In the past, decreasing Hg concentrations have been reported in the liver and kidneys of striped dolphins (*Stenella coeruleoalba*) stranded on the Spanish Mediterranean coast [18], and the same trend was observed in striped dolphins and Risso’s dolphins (*Grampus griseus*) from the Adriatic coast [19]. However, no temporal variation was found in the organs of striped dolphins stranded in the eastern Mediterranean [20], and by collecting global data from peer-reviewed articles and technical reports on total Hg concentrations in the liver of 43 cetacean species, Kershaw and Hall [5] found no apparent changes in reported Hg concentrations over the period 1972–2017. In common dolphins (*Delphinus delphis*) and harbor porpoises (*Phocoena phocoena*) sampled along the French Atlantic coast between 2000 and 2017, Pb concentrations decreased in both species (consistent with the introduction of unleaded gasoline and a general decrease in environmental Pb contamination), whereas Hg concentrations increased significantly in common dolphins but not in harbor porpoises [21]. These results indicate that the role of marine mammals as biomonitors of environmental contamination may be influenced by local species-specific changes, such as those in feeding habits, foraging depths, and/or growth rates, rather than temporal variations in the environmental bioavailability of Hg. Furthermore, although Lee et al. [22] found that declining Hg concentrations in Atlantic bluefin tuna (*Thunnus thynnus*) between 2004 and 2012 reflected reductions in anthropogenic Hg emissions in North America and atmospheric Hg° concentrations in the North Atlantic, declining trends in atmospheric Hg deposition are often not followed by similar trends in metal bioaccumulation in aquatic organisms (e.g., [23,24]). Thus, there is a growing awareness that in addition to possible changes in the feeding behavior of organisms, climate change and its impact on local terrestrial and marine processes and the biogeochemical cycling of the metal could be an important driver of significant changes in MMHg biomagnification along marine food webs [2,25].

The Mediterranean Sea is a semi-enclosed basin that, in addition to receiving atmospheric Hg from anthropogenic activities, forest fires, active volcanoes, and geothermal fields [26,27,28,29], is also influenced by Hg inputs from land-based (rivers and wastewater) and submarine sources (volcanoes, hydrothermal vents, and cold seeps) [1]. Moreover, the basin is one of the most sensitive to climate change [30], and the warming of its waters (20% faster than the global average) and the increase in their salinity, acidity, and stratification affects primary productivity, food webs, and the biogeochemical cycle of Hg [2,31,32,33]. In the Mediterranean region, the frequency of extreme weather events is increasing, with exceptional river flooding and shoreline retreat [34]; furthermore, sea level rise (3.6 ± 0.3 mm year^−1^ in the period 2000–2018) favors the submergence of coastal alluvial plains [35]. As a consequence, along the more than 46,000 km of the Mediterranean coastline, there is an increasing mobilization of legacy Hg, now buried in riverine and marine sediments and released in the past by anthropogenic activities and natural weathering processes [36,37,38]. Taking into account the possible effects of climate change on the biogeochemical cycle of Hg in the different regions of the Mediterranean Sea, this review discusses possible future changes in Hg bioaccumulation in marine mammals.

Since its discovery, the “Mediterranean Hg anomaly” has raised concerns about possible MMHg toxicity to end consumers such as seabirds or pregnant women and children overexposed to seafood [39,40]. For marine mammals, as soon as it was discovered that Se can detoxify Hg and other metals such as cadmium (Cd) and Pb [41], many studies measured Se and Hg concentrations and calculated the Hg:Se molar ratios, and there was relatively less alarm about potential Hg threats (e.g., [3,42,43,44,45]). However, Mediterranean sea mammals are also chronically exposed to and accumulate many other potentially toxic contaminants [46,47,48,49,50], and although it is very difficult to assess, the cumulative biological effects of a complex mixture of persistent contaminants and synergistic effects with other disturbance factors (boat noise, reduced prey due to overfishing, etc.) are undoubtedly more serious than those posed by Hg alone [51]. Therefore, with the aim of contributing to the conservation and management of Mediterranean cetaceans, an update on the possible effects of cumulative stressors on Mediterranean marine mammals is provided, emphasizing the most threatened species and marine areas.

## 2. Mercury Biogeochemistry and Biomagnification in Mediterranean Pelagic Food Webs

The enhanced accumulation of Hg in Mediterranean marine organisms compared to those of other seas is due to the unique geological, morphological, physicochemical, and bio-ecological features of the basin and to thousands of years of anthropogenic impacts. The region is crossed by the Iberian Hg belt (from Almaden in Spain to Monte Amiata in central Italy, Idrjia in Slovenia, and the Aegean Turkey) and contains more than 50% of the global cinnabar (HgS) deposits, which were intensively exploited until a few decades ago. The waters and sediments of the northern Tyrrhenian Sea and the Adriatic Sea received several thousand tons of Hg from past mining and smelting activities and from natural weathering processes on mineralized areas and mine dumps [52,53]. In the Mediterranean Sea, there are many volcanic-associated ecosystems down to depths of 3800 m, which are significant sources of Hg [54,55], and other potential sources could be groundwater discharging directly to the sea, sometimes at great depths [56]. Due to the low exchange with the Atlantic Ocean and other seas, Mediterranean waters have a low renewal rate and receive approximately 37 Ma of Hg per year through wet and dry atmospheric deposition from local and distant anthropogenic (incinerators, cement production, fossil fuel combustion) and natural (forest fire, active volcanoes, fumaroles, and geothermal fields) sources [1,57]. However, with the exception of some coastal areas more impacted by industrial and urban effluents and/or geochemical anomalies, Hg concentrations in open waters and sediments of the Mediterranean Sea are in the same range or only slightly higher than those in the oceans [1,2]. What characterizes the biogeochemical cycling of Hg in the Mediterranean and contributes to the “Hg anomaly” is the enhanced bioavailability and biomagnification of MMHg along food webs [1].

Mercury entering the marine environment has strong affinity for organic matter, chloride, and inorganic sulfides and can form particulates that settle in the sediment where anaerobic bacteria can convert Hg^2+^ in MMHg. Since only anaerobic bacteria and archea are known to methylate the metal, it was long believed that the neurotoxic and bioaccumulative MMHg was mainly produced in the sedimentary environment. However, it was later found that the MMHg accumulating in pelagic fish such as tuna is produced in the upper water column (<1000 m) and reaches maximum concentrations in low-oxygen thermocline waters [58]. Recent oceanographic expeditions suggest that Hg can also be methylated by a microaerophilic and nitrite-oxidizing bacterium (genus *Nitrospina*) that is widespread in oxic sub-surface waters of all oceans, including the Mediterranean [59]. The availability of MMH in sub-surface oceanic waters is confirmed by the stable isotopic signature of Hg in suspended particulates, plankton, and fish [60]. In the stratified Mediterranean water column, MMHg is mainly produced in intermediate low-oxygen waters, where the warm temperature and the re-mineralization of slowly sinking organic materials and fecal pellets favor the activity of methylating bacteria [61]. MMHg concentrations are higher in the western Mediterranean than in the ultra-oligotrophic eastern basin, where low primary productivity and water transparency probably favor the photochemical degradation of MMHg. In any case, in the waters of the Atlantic and Pacific oceans, MMHg represents only a small percentage of the total Hg concentration, while in the waters of the Mediterranean, the percentage is much higher [1]. Furthermore, due to oligotrophy and high nitrogen/phosphorus (N/P) ratios, most of the primary production in the basin is due to very small pico- or nano-phytoplankton cells, which have very high surface-to-volume ratio [62] and absorb and accumulate MMHg at concentrations 5–6 orders of magnitude higher than those in seawater [2]. MMHg has a much longer biological half-life than iHg, so the amounts bioconcentrated by small autotrophs and prokaryotic heterotrophs are transferred to grazing microzooplankton, meso- and macrozooplankton, plankton feeders and carnivorous fish, and finally, to long-lived top predators such as marine mammals. Organisms at each higher trophic level have longer life cycles than their prey and, therefore, bioaccumulate higher concentrations of MMHg. In the Mediterranean macro-zooplankton, for example, the mean MMHg content is more than an order of magnitude higher than in phytoplankton, while iHg concentrations are 2–3 times lower [2,63,64]. The involvement of the microbial food web and very small phyto- and zooplankton organisms makes the Mediterranean pelagic food web longer and more complex, favoring MMHg biomagnification. In addition, most organisms in oligotrophic Mediterranean waters grow more slowly than their Atlantic counterparts and, for the same weight or length, have much higher Hg concentrations because they feed for longer periods on prey with higher MMHg concentrations.

## 3. Mercury Accumulation in Mediterranean Marine Mammals

Despite the general oligo- or ultra-oligotrophic conditions, the Mediterranean basin is characterized by strong gradients in atmospheric precipitation, riverine inputs, nutrient availability, water temperature, and salinity from the north-western to the south-eastern basin. The presence of a variety of marine habitats contributes to a high level of biodiversity with more than 17,000 species, of which about one fifth are considered endemic [65]. During the Messinian crisis (from 5.3 to about 6.0 million years ago), the closure of the connection with the Atlantic Ocean determined the partial desiccation of the basin and the loss of most of the marine fauna. After the reopening of the Strait of Gibraltar, many species of Atlantic organisms, including marine mammals, re-colonized the basin. A total of 28 mammal species are known to occur or have occurred in the Mediterranean Sea [15], although fewer are currently regularly sighted or found stranded along the coasts. Some populations of the threatened and critically endangered monk seal (*Monachus monachus*) survive mainly in the Ionian and Aegean seas and along the coasts of mainland Greece [66]. Among the cetaceans, the most common are the striped dolphin (*S. coeruleoalba*), which prefers the open sea (water depth > 200 m), and the bottlenose dolphin (*Tursiops truncatus*), which is usually sighted in continental shelf waters [67]. The other cetaceans, such as the Risso’s dolphin (*G. griseus*), long-finned pilot whale (*Globicephala melas*), Cuvier’s beaked whale (*Ziphius cavirostris*), sperm whale (*Physeter macrocephalus*), and the only mysticete, the fin whale *Balaenoptera physalus*, are much rarer and occur mainly in the western basin. Only the common dolphin (*D. delphis*) seems to prefer the waters of the eastern Mediterranean [67]. The main feeding and breeding area for cetaceans in the Mediterranean is the Corso–Provencal–Ligurian basin, where the Sanctuary for the Protection of Mediterranean Marine Mammals was established in 2002. Although the French, Ligurian and Tuscan coasts are densely populated and the marine environment is affected by many anthropogenic activities, primary productivity in this region is favored by riverine nutrient inputs (especially from the Rhone in the Gulf of Lion) and local deep-water upwelling. Other important areas for Mediterranean cetaceans are the Alboran Sea, the Campanian and Pontine Archipelagos (Tyrrhenian Sea), eastern Sicily, and the Hellenic Trench [67].

In addition to the environmental bioavailability of persistent contaminants, their bioaccumulation depends on the age, size, diet, physiological conditions (e.g., health and reproductive status), and detoxification and excretion capacity of each individual [68]. During their evolution in the marine environment, seabirds and marine mammals have been exposed to essential and potentially toxic elements and have developed very efficient metabolic pathways to neutralize the toxicity of heavy metals and to accumulate them in very high concentrations in the liver and other organs [12,14,68].

Information on the anatomy-pathology, toxicology, and accumulation of contaminants in marine mammals is usually obtained by analyzing organs and tissues from the stranded individuals. The sampling of skin biopsies from free-ranging animals is another approach that is very useful for pollutant and stable isotope analyses and genetic and toxicological studies [69,70,71]. Although the skin of cetaceans has a turnover rate of several weeks, this tissue is considered a reliable indicator of Hg exposure because it receives through the bloodstream the metal released by demethylation and redistribution processes occurring in the liver [72]. Thus, total Hg concentrations in the skin are usually significantly correlated with those in internal organs and can be used to estimate the metal content in the liver, kidney, or brain [73,74].

Diet is the most important driver of Hg accumulation in marine apex predators, and by measuring stable Hg isotopes in Pacific bluefin tuna (*Thunnus orientalis*), Madigan et al. [75] found that increased mesopelagic feeding decreased growth and increased MMHg levels. Thus, potential changes in diet and/or growth of odontocetes may alter Hg bioaccumulation independent from variations in the environmental availability of the metal [76]. In Mediterranean cetaceans, for example, Aznar et al. [77] reported a change in feeding behavior in striped dolphins stranded along the Spanish coast.

After rapid growth in weight and length, the size of the cetaceans remains almost constant at a certain age, and adults consume more food and larger prey with higher MMHg content. Thus, their Hg load increases disproportionately compared to that of young individuals, where the lower metal input is “diluted” by the growing body. Therefore, to make reliable comparisons of Hg bioaccumulation in different cetacean populations, assuming a fairly similar diet, it is imperative to compare data from individuals of the same age or size, or at least from pools of animals belonging to the same growth stage (e.g., [78]). In general, statistically significant differences between total Hg concentrations in the organs of male and female dolphins have not been reported; however, higher levels of Hg have sometimes been measured in the organs of females, especially those that are pregnant or lactating, who are likely to require greater amounts of energy and food [19].

Since the 1990s, many data on Hg concentrations in organs and tissues of *S. coeruleoalba* have been published. Although the decomposition state of stranded animals sometimes does not allow the determination of the length or collection of some organs, most studies provide information on the age or size of stranded individuals but often report the mean Hg concentration in each organ of all individuals. In order to make more reliable comparisons among the literature data on Hg bioaccumulation in Mediterranean striped dolphins, Table 1 reports only the mean or range of Hg concentrations for a given age or length class. In general, *S. coeruleoalba* in the Mediterranean grow approximately 10% shorter than in the Pacific, and individuals < 120 cm in length are usually classified as calves and those <190 cm as subadults [79]. However, although no sexual dimorphism was found in 125 striped dolphins stranded along the northern Mediterranean coasts of Spain and in the Ligurian and northern Tyrrhenian seas [66], according to Calzada et al. [79], females are smaller than males and may reach sexual maturity at 187–188 cm.

The mercury concentrations in the organs and tissues of striped dolphins increase with age and are highly variable even among individuals from the same population of similar length (Table 1). The liver is the organ most involved in the demethylation of MMHg and the compartmentalization of tiemannite crystals and consequently accumulates the highest levels of the metal. Furthermore, in the liver and other organs such as the kidney, most of the metal is present as iHg, whereas in the muscle and heart, organic MMHg predominates [86]. Because of its very high total Hg content, the liver has usually been considered the best indicator of long-term changes in Hg bioavailability in marine mammals. However, by using Hg isotopes to understand the metabolic mechanisms and internal dynamics of Hg in the biota, Li et al. [72] have recently shown that the content of δ^202^Hg in the liver and other organs can vary between life stages of cetaceans due to demethylation and redistribution processes, while the composition of Hg isotopes in the blood is almost constant. Thus, although blood has rarely been analyzed for total Hg, they suggested that the latter may be a more appropriate indicator of spatio-temporal changes in Hg availability.

In general, no relationships have been found between Hg bioaccumulation in striped dolphins and different stranding locations; however, as reported in some studies (e.g., [69,81]), the data summarized in Table 1 indicate an increased bioaccumulation of Hg in cetaceans stranded in the NW Mediterranean, Ligurian, and North Tyrrhenian seas (i.e., the main feeding grounds for most Mediterranean cetaceans, with the highest bioavailability of MMHg) [1,2]. In the same regions, total Hg levels in skin biopsies or skin samples of *S. coeruleoalba* were reported to be approximately 2–4 µg g^−1^ wet weight [69,87].

Bottlenose dolphin (*T. truncatus*) and Risso’s dolphin (*G. griseus*) are two other species frequently stranded on Mediterranean coasts. In general, the patterns of Hg accumulation in the tissues of these dolphins mirror those of striped dolphins; however, because of their larger body length and mass and different habitats and diets, these species have higher Hg concentrations [19,20,85,87]. For instance, the mean total Hg concentrations (µg g^−1^ wet weight) in the livers of several specimens of *G. griseus*, *T. truncatus,* and *S. coeruleoalba* of different ages stranded along the Croatian coast of the Adriatic Sea between 1995 and 2014 were 807 ± 806, 199 ± 322, and 112 ± 139, respectively [19]. Much less data are available for the other Mediterranean marine mammal species; 33.5 and 256 µg g^−1^ wet weight were measured in the liver of a common dolphin and a long-finned pilot whale stranded on the Corsican coast, respectively [88]; 260 µg g^−1^ wet weight in the liver of an adult sperm whale from the Adriatic coast [43]; and 64.5 µg g^−1^ wet weight in that of a Cuvier’s beaked whale from the Ligurian Sea [86]. The fin whale (*B. physalus*), the largest cetacean and the only mysticete regularly found in the Mediterranean Sea, accumulates much lower concentrations of Hg in its organs and tissues compared to other cetaceans (often <1.5 µg g^−1^ wet weight), reflecting its different diet and the trophic levels of its prey [86]. The Mediterranean monk seal (*M. monachus*) from the Ionian and Aegean Seas also accumulates relatively low concentrations of Hg, probably because, unlike cetaceans, it can eliminate the metal by shedding its hairs. Formigaro et al. [89] found that the mean levels in the liver and kidney of 59 deceased individuals (4.09 ± 7.57 and 1.45 ± 2.28 µg g^−1^ wet weight, respectively) were much lower than those previously reported for common (*Phoca v. vitulina*), ringed (*Pusa hispida*), grey (*Halichoerus g. grypus*), and hooded seals (*Cristophora cristata*) from other seas.

Concerning the Hg content in the skin of Mediterranean cetaceans, Pinzone et al. [90] analyzed 66 samples from *B. physalus*, 29 from *G. melas*, and 19 from *P. macrocephalus*, freely ranging in the north-western Mediterranean Sea, and measured mean concentrations (0.2 ± 0.05, 8.2 ± 2.3, and 2.7 ± 0.9, respectively) much higher than those measured in skin samples of related cetacean species from the Atlantic and other oceans. To evaluate the role of the trophic behavior of each species in Hg accumulation, they also analyzed stable isotopes of C, N, and S and found that prey type selection was the main factor influencing Hg bioaccumulation, with higher levels in generalist species such as *G. melas* than in *P. macrocephalus*, which feeds mainly on cephalopods.

## 4. Mercury–Selenium Interactions

In general, iHg is poorly absorbed in the gastrointestinal tract and is readily excreted in urine and feces, but marine mammals ingest MMHg with their prey, which is efficiently absorbed, enters the liver and bloodstream, binds to cysteine (mimicking methionine), and crosses cell membranes via amino acid transporters [91]. The rapid flow of MMHg throughout the body of marine mammals is evidenced by the similar isotopic composition of its end-members in the kidney, muscle, heart, and brain [92]. Unlike iHg and other heavy metals, MMHg has little affinity for metallothioneins and is detoxified by demethylation mediated by Se-containing biomolecules. The resulting labile iHg compounds bind to Se to form insoluble crystals of tiemannite or are redistributed from the liver to other organs and tissues [92]. Although the occurrence of chemically inert tiemannite crystals was reported in the 1990s only in the liver of marine mammals (e.g., [12,13,14]), subsequent studies using micro X-ray fluorescence imaging and micro X-ray diffraction revealed their occurrence in other organs and tissues [93]. However, in older cetaceans, MMHg demethylation occurs mainly in the kidney and especially in the liver [92], and in bottlenose dolphins, these organs have been shown to accumulate tiemannite crystals that are much larger and more nodular than those in the brain or other tissues [94].

Young Odontoceti have a different metabolism and isotopic composition of Hg than adults [92]. In fact, demethylation is not activated in juveniles and their tissues contain higher percentages of MMHg compared to the total Hg content. After the dietary shift during weaning, adult cetaceans are exposed to increasing MMHg inputs through the diet and use Se biomolecules for the detoxification process and the formation of tiemannite crystals. Thus, concentrations of iHg, MMHg, and Se in organs and tissues of cetaceans vary with life stages. For example, Li et al. [92] measured Se concentrations up to 30 times higher in the liver of older whales than in younger ones, and while the molar ratio of Hg:Se in adults is usually close to unity, the values are much higher in young specimens.

Selenium in mammals is required for the synthesis of amino acids and hormones and is involved in many other cellular functions such as immunity and redox homeostasis; moreover, this essential element has very narrow ranges between essential and toxic levels [95]. In older toothed whales and dolphins, the involvement of this element in MMHg detoxification contributes to the depletion of their biological Se pool, the reduction of brain Se-cysteine levels, and an increased susceptibility to infectious diseases and autoimmune disorders [96]. For this reason, the liver of stranded cetaceans with infectious diseases has often been found to contain higher Hg and Se concentrations than the liver of animals that died from physical trauma [5]. Furthermore, Li et al. [92] hypothesized that reduced availability of Se in adult whales with very high liver Hg concentrations should increase their systemic circulation of iHg. The data on total Hg concentrations summarized in Table 1 indicate that this redistribution is very likely in the organs of older *S. coeruleoalba* individuals stranded along the coasts of the Ligurian Sea.

The liver is the target organ and plays a major role in the detoxification of potentially toxic elements and in the homeostatic regulation of essential ones; therefore, in marine mammals, it also accumulates the highest concentrations of Zn, Cu, and other trace elements. Among the heavy metals, Cd tends to accumulate particularly in the kidney (which binds Cd metallothioneins) and Pb in bone [8]. The increased exposure of marine mammals to metals and metalloids may be associated with neurological and other toxic effects [97]. However, the concentrations of most essential trace elements in Mediterranean whales and dolphins appear to be under homeostatic control [43,69,80,86,89], and those of Cd, a non-essential and potentially toxic element, are usually lower than those reported for cetaceans from other seas. As a rule, higher Cd concentrations in the kidney and liver of marine mammals are associated with squid-rich diets, and the highest levels are usually reported for those living in polar and subpolar seas. In cold and highly productive Antarctic waters, for instance, the metal is rapidly regenerated and transferred along trophic food webs through phyto- and zooplankton [98]. Liver and kidney Cd concentrations in Antarctic marine birds and mammals are much higher than in Mediterranean species, and although levels are often well above the toxic thresholds established for humans, Antarctic animals appear to tolerate the increased natural availability of Cd [98].

## 5. Climate Change and Future Trends of Mercury Bioavailability in the Mediterranean Sea

The remarkable Hg burden in Mediterranean cetaceans is mainly due to their trophic position and the unique physico-chemical and bio-ecological features of the semi-enclosed and oligotrophic basin, which has been affected by natural and anthropogenic Hg sources since ancient times. Therefore, to predict future trends of Hg bioaccumulation in marine mammals, the impacts of human activities and climate change on atmospheric deposition and continental inputs of the metal should be considered. Moreover, the Mediterranean Sea is very sensitive to global change, and the warming (20% faster than the global average) is likely to increase seawater stratification and oligotrophy [33,99], with significant changes in biotic communities, methylation, demethylation and biomagnification of MMHg in pelagic food webs.

Atmospheric depositions of Hg in the Mediterranean are not well known, especially those from natural sources such as forest fires, volcanic and geothermal activities, and natural out-gassing of Hg° in cinnabar mineralized areas, abandoned Hg mining, and smelting areas [100]. However, atmospheric precipitation and anthropogenic emissions of the metal are decreasing in the region [6], and human activities are estimated to contribute only 20–25% of total wet and dry Hg deposition [29]. In addition, warming and stratification of surface waters are likely to increase the escape of Hg° to the atmosphere [2]. Recent high-spatial-resolution datasets suggest that in coastal oceans, rivers are about three times greater sources of Hg than atmospheric deposition [101]. Since the Mediterranean basin is almost landlocked, with more than 46,000 km of coastline, continental Hg inputs are probably even higher and are likely to increase due to the greater frequency of extreme weather events and flooding. While the sea level in the Mediterranean is rising by approximately 3.6 mm year^−1^ [102], several coastal areas are subsiding, and the intensification of storms is likely to increase coastal erosion and the mobilization of legacy Hg accumulated in many estuaries and coastal environments [2].

Recent ocean–atmosphere–land models, taking into account the current knowledge on the atmospheric chemistry of Hg, its isotopic composition in seawater, and the estimated higher riverine inputs [103,104], predict that rising water temperatures will decrease the solubility of the metal in seawater and promote the escape of Hg°, especially in marine regions where the wind speed will not decrease [104]. Warming and stratification of the P-limited and oligotrophic waters of the Mediterranean are likely to reduce phytoplankton production, Hg scavenging, and availability to methylating microorganisms in deeper, low-oxygen waters. Warmer and less dense surface waters will increase the metabolism of marine organisms and are likely to promote further development of very small planktonic species, and models predict that smaller zooplankton organisms should reduce the MMHg biomagnification [104]. Although global projections for mid-latitude seas seem to exclude significant increases in bioavailability and biomagnification of MMHg, some specific physico-chemical and biological characteristics of the Mediterranean Sea could favor Hg bioaccumulation in marine mammals. For instance, the increased mobilization of Hg in coastal and shelf environments, possible changes and lengthening of pelagic food webs, and overfishing may contribute to dietary shifts and lower growth rates of long-lived odontocetes, increasing their Hg bioaccumulation.

## 6. Possible Cumulative Effects of Hg and Persistent Organic Pollutants (POPs)

Knowledge of the biological effects of Hg accumulation and the concomitant Se deficiency in marine mammals is still insufficient, but it seems even more important to study possible synergistic or additive effects of exposure to Hg, POPs and other environmental pollutants. In fact, many legacy compounds and some of those of emerging toxicological interest are widespread in the Mediterranean and can persist in the marine environment for years; they are lipophilic and bioaccumulate and biomagnify along food webs. Due to their diet, long lifespan, fat blubber, and melon tissue, marine mammals accumulate the highest concentrations of hydrophobic compounds of all marine organisms. Thus, despite restrictions and bans under the Stockholm Convention, Mediterranean species are among the most exposed in the world to many harmful chemicals such as chlorinated compounds, polycyclic aromatic hydrocarbons (PAHs), pesticides, and brominated flame retardants [46,47,48,49]. These chemicals can cause neurological, immunological, endocrine, cardiovascular, pulmonary, and reproductive disorders [17,105]. In the case of the Mediterranean monk seals, for instance, while total Hg concentrations in their liver and kidneys [89] are much lower than those of odontocetes from the same marine areas (Table 1), POP concentrations in their blubber are in the same range or higher than those of dolphins (Table 2). The load of organic pollutants in the Eastern Mediterranean monk seal is higher than in the Western Sahara subpopulation and in the Hawaiian monk seal (*Monachus schauinslandi*). Moreover, while their HCB and DDT concentrations were found to be decreasing, those of PCBs did not show a similar trend and were above the established threshold in approximately 70% of the specimens analyzed [49].

In general, concentrations of organic pollutants in marine mammal organs and tissues are highly variable and often no statistically significant differences are observed between different sexes or age classes (Table 2). The higher levels of POPs found in males have usually been attributed to the fact that males accumulate contaminants in their tissues continuously throughout their lives, while females have the potential to transfer contaminants (up to 90%) to their offspring during pregnancy and lactation [109]. Due to the variability of the data, it is often difficult to identify statistically significant differences in environmental pollution levels between different stranding sites in the Mediterranean. However, as found for total Hg concentrations (Table 1), higher levels of PCBs, DDTs, PAHs, and HCB were often reported for dolphins stranded in the northern Tyrrhenian Sea and the NW Mediterranean (Table 2). In general, in most studies, the value of the DDTs/PCBs ratio is <1, probably indicating a predominant industrial source of organic pollutants, and that of the pp’-DDE/DDT ratio suggests little or no release of new pesticides into the Mediterranean environment. However, it is difficult to assess temporal changes in the bioavailability of organic pollutants from the available data, as most studies usually report their mean concentration in tissues and organs of stranded cetaceans over long time intervals (Table 2). By analyzing historical data (1998–2016) from tissues of *S. coeruleoalba* stranded in the NW Mediterranean, Dron et al. [107] found a slow decreasing trend in pollutant concentrations since 1988, but from 2010 onwards, the levels of several compounds seemed to plateau at levels still above the estimated toxicological threshold for cetaceans. A quite similar temporal trend was reported for most of the long-chain perfluoroalkyl substances (PFAS) in the livers of 48 adult striped dolphins stranded on the Catalan coast of Spain, with increasing concentrations from 1990 to 2009 and a subsequent stabilization of levels from 2014 to 2021 [111]. Thus, although concentrations of some legacy POPs in organs and tissues of Mediterranean mammals are lower and not as extreme as 20–30 years ago, they still seem capable of causing toxicological effects. Cetacean populations living in the densely populated and industrialized regions surrounding the Pelagos Marine Sanctuary seem to be particularly exposed. Already in 2013, by analyzing concentrations of organochlorines and flame retardants, diagnostic markers of exposure, and those of genetic erosion in skin biopsies of *S. coeruleoalba*, it was found that dolphins from the North Tyrrhenian and NW Mediterranean (which also have the highest total Hg concentrations; Table 1) had the highest levels of organic pollutants and suffered the most toxicological stress. Moreover, as discussed above for continental Hg inputs and the impact of climate change on its remobilization, it has been suggested that the NW Mediterranean basin is affected by the remobilization of banned POPs released in the past from agricultural, industrial, or urban sources and accumulated in soils, the Rhone, and other river sediments [107,112].

Most studies on the toxic effects of pollutants in marine mammals have focused on immunotoxicity, which is well known for POPs and metals such as Hg and Cd. However, it cannot be excluded that contaminants of emerging interest (e.g., pharmaceuticals, personal care products, industrial compounds) and micro- and nanoplastic particles [17,46,48,51,111] may also contribute to increased susceptibility to disease, bacterial infection, and parasitism. For instance, the ingestion of microplastics can have direct biological effects such as internal abrasion, but due to their high specific surface area, the smaller fragments can also accumulate metals and organic contaminants to levels orders of magnitude higher than in seawater and can also act as vectors for pathogens [113].

The biological effects of simultaneous exposure to a range of organic and inorganic pollutants that may interact in synergistic, additive, or antagonistic ways are difficult to assess. Moreover, the resilience and responses of marine mammals may be exacerbated by the concurrent effects of other anthropogenic disturbances and climate-related stressors. Most studies on the biological effects of environmental pollutants are based on the exposure of blood or skin cells (non-destructively sampled from living animals) to POPs or metals and assess early adverse outcomes [17,51]. Thus, most reported biological effects occur at the level of molecular and cellular organization and are rather non-specific (e.g., up- or down-regulation of a gene, inhibition of certain receptors or cellular pathways). However, information at higher levels of biological organization, such as tissues, organs, or individuals, would be needed. To elucidate pollutant-induced effects on the immune, endocrine, neurological, reproductive, and developmental systems, Schaap et al. [17] propose to interpret data on pollutant concentrations in marine mammal organs and tissues using the physiologically based toxico-kinetic model used for human risk assessment and to translate molecular and cellular perturbation data into effect data detected in organs, organisms, or populations.

## 7. Management Priorities for Mediterranean Marine Mammals

**Figure 1:**
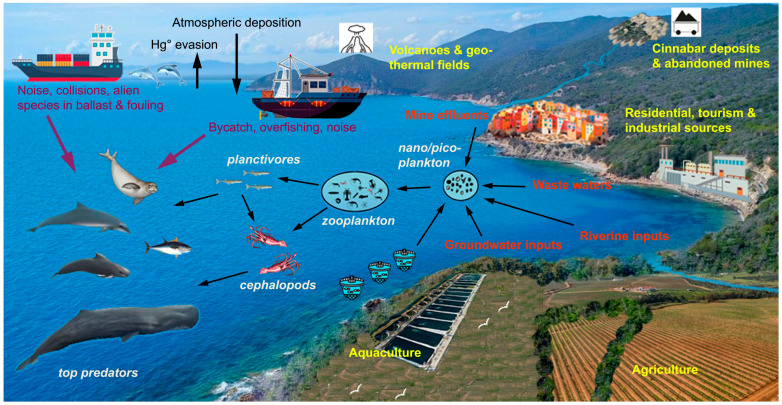
Sources and biomagnification of Hg and POPs along pelagic food webs and major threats to marine mammals in the Mediterranean Sea.

There are 92 species of cetaceans in the world, and the number of threatened species (critically endangered, endangered, or vulnerable) has increased from 15% in 1991 to 26% in 2021. The species listed as threatened tend to have narrow geographic ranges, live in fluvial or coastal habitats, and are impacted by various anthropogenic activities [114]. According to a comprehensive review of the current global risks to marine mammals [51], the main threats are incidental capture, environmental pollution, direct harvest, collisions, and noise pollution from vessels. Although the risk for the same species varies considerably in different geographical areas, odontocetes, and especially Delphinidae, face the greatest number of threats globally, and a common and widespread species such as *T. truncatus* is affected by the widest range of threats [51]. As the Mediterranean Sea is a semi-enclosed and densely populated basin, its odontocete populations are undoubtedly among the most exposed to cumulative anthropogenic impacts. As shown by the data reported above, the bioaccumulation of Hg and POPs unquestionably constitute a very significant impact, and equally important are those of fishing, maritime transport, aquaculture, tourism, urbanization, and coastal habitat degradation (Figure 1). Moreover, the Mediterranean is a hot-spot for climate change, which is likely to have a negative effect on primary productivity and the availability of food resources for final consumers. Warming waters facilitate the colonization and establishment of exotic species entered from the Suez Canal, or accidentally or intentionally introduced through the aquarium trade or through ballast water and fouling communities on ships’ hulls. Currently, approximately 1000 alien species have been identified in the Mediterranean Sea, and some of them are already threatening the local biotic communities, which include more than 3000 endemic species [115].

Due to the direct or indirect exposure to a number of cumulative impacts, there is an urgent need for more stringent conservation strategies for Mediterranean marine mammals, especially in the main feeding and breeding area: the Pelagos Sanctuary. Several papers have already highlighted the threats to cetaceans in this protected area and the need to maintain strict monitoring and surveillance activities (e.g., [46,48,107,111,116,117]). In addition, Azzellino et al. [118], who modeled the spatio-temporal distribution and habitat preference of seven cetacean species regularly observed in the Sanctuary, found temporal variability in habitat use by fin whale, sperm whale, and striped dolphin populations. Further significant changes in the bioaccumulation of Hg and POPs are likely to be promoted by the effects of climate change on the physico-chemical characteristics of marine waters, their productivity, pelagic food webs, and prey availability for cetaceans. In this context, the European Union’s Marine Strategy Directive (2008/56/EC; http://data.europa.eu/eli/dir/2008/56/2017-06-07 (accessed on 1 July 2024).) mandates the protection of marine ecosystems and biodiversity through concrete measures at the international level to reduce all pressures resulting from human activities. While several mitigation measures can already be applied to fishing activities and maritime traffic [https://faolex.fao.org/docs/pdf/mul217150.pdf (accessed on 1 July 2024); https://portals.iucn.org/library/sites/library/files/documents/2008-042-1.pdf#page=160 (accessed on 1 July 2024)], in the case of environmental contaminants (legacy or of emerging interest), there are rather strong policies and regulations in Europe for their production and use. To reduce the transfer of persistent pollutants to marine ecosystems, freshwater treatment could be enhanced. Several methods have been developed to remove persistent pollutants from water and wastewater including reverse osmosis, membrane filtration, adsorption, coagulation, flocculation, and nanotechnology [119]. However, these approaches often have excessive costs, low efficiencies, and can produce toxic residues [105]. Thus, while waiting for improved technological approaches capable of removing contaminants (including microplastics) with sustainable costs and environmentally friendly methods, traditional bioremediation with microorganisms, biofilms, macrophytes, and constructed wetlands seem to be the more suitable approaches for now (e.g., [120,121,122]). However, the introduction of even more stringent policies and regulations to further reduce the production and use of persistent environmental contaminants would undoubtedly be the most effective measure to protect marine and terrestrial ecosystems.

## 8. Conclusions

Odontocetes living in the Mediterranean Sea have the highest concentrations of mercury, often at levels above those that could cause potential adverse effects. This review explains how the enormous bioaccumulation of the metal in long-lived and carnivorous whales and dolphins is due to specific features of this semi-enclosed basin, which has been influenced by geogenic and anthropogenic sources for thousands of years. Basically, although Hg concentrations in Mediterranean open waters and sediments are in the same range as in other seas, the enhanced production of MMHg and its very efficient uptake by pico- and nano-plankton cells (which dominate autotrophic production in the oligotrophic and phosphorus-limited Mediterranean waters) are responsible for the enhanced transfer of MMHg along food webs up to the final consumers. This process is further facilitated by longer and more complex pelagic food webs and by the fact that most organisms in the oligotrophic Mediterranean grow more slowly than their counterparts in other marine environments.

The basin is a hotspot for climate change, and although the latest ocean–atmosphere–land models seem to rule out significant increases in the bioavailability of MMHg in mid-latitude seas, it is not easy to predict what will happen in the Mediterranean. If, on the one hand, the warming of the water decreases the solubility of Hg° and favor its escape to the atmosphere, on the other hand, the increasing frequency of extreme weather events is likely to enhance the mobilization of legacy Hg accumulated in soils, riverine, and coastal environments. In addition, the effects of overfishing, climate change, and alien organisms on the composition of Mediterranean biotic communities will probably contribute to dietary shifts and lower growth rates of odontocetes, increasing Hg accumulation.

Many adult marine mammals have acquired the ability to demethylate MMHg with Se-containing biomolecules and store the metal in the liver and other organs as insoluble and non-toxic HgSe crystals. The detoxification process is not activated in juveniles, so the levels and relative proportions of iHg, MMHg, and Se in the liver and the other organs of marine mammals vary with life stage. The liver is the target organ and accumulates the highest concentrations of total Hg, but contrary to what was previously thought, this organ is not the best indicator of changes in the environmental bioavailability of the metal. Recent studies on the internal dynamics and metabolism of Hg isotopes indicate that the content of δ^202^Hg in the liver and other organs varies during life stages due to demethylation and redistribution processes. Blood Hg isotopes are almost constant, and although rather neglected in previous biomonitoring surveys, total blood Hg is probably a more reliable indicator of spatio-temporal changes in metal bioavailability for marine mammals.

Selenium is an essential element for mammals, performing numerous biochemical and cellular functions; therefore, its involvement in the detoxification of MMHg in adult cetaceans depletes its biological pool and increases susceptibility to autoimmune and infectious diseases. A further and probably more serious threat to Mediterranean cetaceans is posed by the concomitant exposure to Hg and many legacy POPs, micro- and nanoplastics, and contaminants of emerging interest. The load of Hg and POPs is particularly high in cetaceans living in the NW Mediterranean and the northern Tyrrhenian Sea, the main feeding and breeding area for several species, where the Mediterranean Marine Mammals Sanctuary was established in 2002. Thus, in proposing possible approaches to assess the biological implications of the simultaneous exposure of cetaceans to organic and inorganic contaminants, this review emphasizes, in line with the provisions of the EU Marine Strategy, the rigorous application of available mitigation measures for fishing activities and maritime traffic to reduce the pressure of human activities on cetacean populations. With regard to the impact of persistent environmental contaminants, the most effective measure appears to be to further reduce their production and use, as currently available technological approaches for their removal are too expensive and cannot be applied.

## Figures and Tables

**Table 1:** Total Hg concentrations (µg g^−1^ wet weight; range, mean ± SD or single measure) in organs and tissues of striped dolphins *S. coeruleoalba* stranded along the Mediterranean coasts. Data are reported by age class (<120 cm, calves; <187–190 cm, subadults; adults). For comparison, dry weight data from references [80,81,82] were converted to wet weights using the conversion factor of 0.25 [83]. ♀ = female; ♂ = male.

Sampling Area	Year	N of Samples	Length (cm)	Liver	Kidney	Muscle	Lung	Brain	Ref.
Gibraltar Str. (Southern Spain)	2012–2013	1	95	1.5	0.79	14.8			[80]
		5	121–184	3.9–88.3	2.1–25.3	1.3–16.3			
		4	200–220	63–270	8.8–26.5	3.9–25.3			
Southern Spain	2009–2015	2	<95	2.5 ± 1.0	0.59 ± 0.55	1.45	0.66	0.59 ± 0.46	[78]
		26	<187 ♀; <190 ♂	27.2 ± 32.3	5.4 ± 4.1	1.9 ± 1.1	1.5 ± 1.7	1.02 ± 0.46	
		25	>187 ♀; >190 ♂	241 ± 158	14.2 ± 7.3	14.1 ± 9.3	13.0 ± 8.3	22.8 ± 24.9	
NW Mediterranean Ligurian Sea	1986–1990	3–4	98–110	3.2–5.3	1.3–2.5	1.3–1.5	0.50–1.25	0.75–1.00	[81]
		3–7	159–188	56.3–178.3	7.8–16.3	3.3–14.3	2.5–6.8	4.0–5.8	
		5–7	193–226	33.0–748	10.5–40.8	19.0–65.0	46–112	8.8–47	
Sicily Channel	2000–2009	3–4	85–110	2.1–22.5	0.1–5.6	0.1–0.3	0.60–1.90		[82]
		3	131–182	5.5–24.4	2.5–5.9	1.3–3.4	0.45–2.82		
		1	190	114	0.1	4.2	17.8		
Apulian coasts	1987	5	190–208	189 ± 28	10.3 ± 2.2	10.9 ± 2.5	28.7 ± 14.2	13.9 ± 6.0	[84]
Eastern Adriatic Sea	2002	5	188–207	182 ± 91.6	12.6 ± 7.3	16.5 ± 13.9			[85]
Israel coasts	1993–2001	1	102	1.4	1.9	0.54			[76]
		5	187–197	26–550	1.9–27	2.3–21			

**Table 2:** Concentrations of organic pollutants (ng g^−1^, lipid weight; mean ± SD, when available) in the blubber of marine mammals stranded along the Mediterranean coasts during the last two decades (IM = immature, ♀ = adult female; ♂ = adult male).

Sampling Area	Period (Year)	Species	Sample (n per Age)	ƩPCBs	ƩDDTs	ƩPAH (n)	HCB	ƩHCH	Ref.
Ionian/Aegean Seas	1995–2013	*Monachus monachus*	31 IM	59,500	55,900	(14) 724	239		[49]
			11 ♀	240,000	171,000	(14) 549	324		
			5 ♂	36,000	47,500	(14) 231	180		
SE Spain	2011–2018	*Stenella coeruleoalba*	12 IM	4340 ± 7090	3865 ± 4628		25.9 ± 28.1	131 ± 237	[106]
			6 ♀	2206 ± 1535	2120 ± 1593		15.9 ± 21.1	75.4 ± 93.5	
			6 ♂	13,361 ± 18,208	9788 ± 15,030		41.5 ± 67.1	242 ± 533	
		*Tursiops truncatus*	8 all	6106 ± 5610	2266 ± 1739		18.6 ± 12.3	73.4 ± 58.8	
SE Spain	2011–2018	*S. coeruleoalba* *T. truncatus*	34 all			(16) 100 ± 59			[47]
			8 all			(16) 109 ± 44			
French Mediterranean coast	1988–2000	*S. coeruleoalba*	5 all	45,200	————				[107]
	2000–2003		3 all	69,978	3108				
	2003–2009		33 all	37,460	17,036				
	2010–2016		45 all	21,058	10,777				
French Mediterranean coast	2010–2016	*S. coeruleoalba* *T. truncatus*	42 all			(16) 1020			[108]
			5 all			(16) 981			
Italian coasts	1998–2021	*Grampus griseus*	2 IM	137,500 ± 193,070	71,050 ± 69,950		370 ± 380		[109]
			10 ♀						
			8 ♂						
Tyrrh./Ligurian Seas	2015–2016	*S. coeruleoalba*	5 ♀	1360					[110]
			5 ♂	2590					

## Data Availability

Data are contained within the article.
